# Characterization of *HNRNPA1* mutations defines diversity in pathogenic mechanisms and clinical presentation

**DOI:** 10.1172/jci.insight.148363

**Published:** 2021-07-22

**Authors:** Danique Beijer, Hong Joo Kim, Lin Guo, Kevin O’Donovan, Inès Mademan, Tine Deconinck, Kristof Van Schil, Charlotte M. Fare, Lauren E. Drake, Alice F. Ford, Andrzej Kochański, Dagmara Kabzińska, Nicolas Dubuisson, Peter Van den Bergh, Nicol C. Voermans, Richard J.L.F. Lemmers, Silvère M. van der Maarel, Devon Bonner, Jacinda B. Sampson, Matthew T. Wheeler, Anahit Mehrabyan, Steven Palmer, Peter De Jonghe, James Shorter, J. Paul Taylor, Jonathan Baets

**Affiliations:** 1 Translational Neurosciences, Faculty of Medicine and Health Sciences, and; 2 Laboratory for Neuromuscular Pathology, Institute Born-Bunge, University of Antwerp, Wilrijk, Belgium.; 3 Department of Cell and Molecular Biology, St. Jude Children’s Research Hospital, Memphis, Tennessee, USA.; 4 Department of Biochemistry and Biophysics, Perelman School of Medicine, University of Pennsylvania, Philadelphia, Pennsylvania, USA.; 5 Department of Biochemistry and Molecular Biology, Sidney Kimmel Medical College, Thomas Jefferson University, Philadelphia, Pennsylvania, USA.; 6 Medical Genetics, University of Antwerp and Antwerp University Hospital, Edegem, Belgium.; 7 Neuromuscular Unit, Mossakowski Medical Research Centre, Polish Academy of Sciences, Warsaw, Poland.; 8 Neuromuscular Reference Centre, University Hospitals St-Luc, University of Louvain, Brussels, Belgium.; 9 Department of Neurology, Donders Institute for Brain, Cognition and Behaviour, Radboud University Medical Center, Nijmegen, Netherlands.; 10 Human Genetics Department, Leiden University Medical Center, Netherlands.; 11 Stanford Center for Undiagnosed Diseases, Stanford University, Stanford, California, USA.; 12 Department of Neurology, School of Medicine, University of North Carolina at Chapel Hill, Chapel Hill, North Carolina, USA.; 13 Neuromuscular Reference Centre, Department of Neurology, Antwerp University Hospital, Wilrijk, Belgium.; 14 Howard Hughes Medical Institute, Chevy Chase, Maryland, USA.

**Keywords:** Genetics, Neuroscience, Cell stress, Neurological disorders

## Abstract

Mutations in *HNRNPA1* encoding heterogeneous nuclear ribonucleoprotein (hnRNP) A1 are a rare cause of amyotrophic lateral sclerosis (ALS) and multisystem proteinopathy (MSP). hnRNPA1 is part of the group of RNA-binding proteins (RBPs) that assemble with RNA to form RNPs. hnRNPs are concentrated in the nucleus and function in pre-mRNA splicing, mRNA stability, and the regulation of transcription and translation. During stress, hnRNPs, mRNA, and other RBPs condense in the cytoplasm to form stress granules (SGs). SGs are implicated in the pathogenesis of (neuro-)degenerative diseases, including ALS and inclusion body myopathy (IBM). Mutations in RBPs that affect SG biology, including FUS, TDP-43, hnRNPA1, hnRNPA2B1, and TIA1, underlie ALS, IBM, and other neurodegenerative diseases. Here, we characterize 4 potentially novel HNRNPA1 mutations (yielding 3 protein variants: *321Eext*6, *321Qext*6, and G304Nfs*3) and 2 known HNRNPA1 mutations (P288A and D262V), previously connected to ALS and MSP, in a broad spectrum of patients with hereditary motor neuropathy, ALS, and myopathy. We establish that the mutations can have different effects on hnRNPA1 fibrillization, liquid-liquid phase separation, and SG dynamics. P288A accelerated fibrillization and decelerated SG disassembly, whereas *321Eext*6 had no effect on fibrillization but decelerated SG disassembly. By contrast, G304Nfs*3 decelerated fibrillization and impaired liquid phase separation. Our findings suggest different underlying pathomechanisms for HNRNPA1 mutations with a possible link to clinical phenotypes.

## Introduction

Heterogeneous nuclear ribonucleoprotein (hnRNP) A1 is a member of a large class of RNA-binding proteins (RBPs) that assemble with RNA to form ribonucleoproteins (RNPs). hnRNPs are evolutionarily conserved and are primarily localized to the nucleus, where they function in pre-mRNA splicing, mRNA stability, miRNA maturation, regulation of transcription and translation, and telomere biogenesis (1–7). Mutations affecting several RBPs, including FUS, TDP-43, hnRNPA1, hnRNPA2B1, matrin-3, and TIA1, are linked to amyotrophic lateral sclerosis (ALS), frontotemporal dementia (FTD), multisystem proteinopathy (MSP), and hereditary inclusion body myopathy (hIBM) phenotypes (8, 9).

Missense mutations in the prion-like domain (PrLD) of hnRNPA1 (p.D262V/N and p.N267S) are linked to ALS (MIM 615426) and MSP3 (MIM 615424) (10, 11). Additional ALS-linked mutations in hnRNPA1 are also in the PrLD (p.G264R) (12) or the nuclear localization sequence (p.P288S/A) (13, 14). The p.D262N mutation in *HNRNPA1* was also reported in 2 families with IBM with a pure muscular phenotype, illustrating the heterogeneity of consequences that arise from *HNRNPA1* mutations. Several modest-sized genetic screenings failed to identify further *HNRNPA1* mutations in 2485 patients with ALS, FTD, MSP, hIBM, or other myopathies, suggesting that disease-linked mutations in *HNRNPA1* may be rare (15–19).

Stress granules (SGs) are at the nexus of RBP pathology and neurodegenerative diseases (20–27). SGs are cytoplasmic membrane-less organelles that contain RNA and proteins, including several RBPs connected with neurodegeneration (22). SGs mostly contain mRNA transcripts, with approximately 80% of the SG consisting of mRNAs from essentially every expressed gene (28, 29). SGs also specifically contain small ribosomal subunits, translation initiation factors (eIF3, eIF4E, eIF4G), hnRNPs, and other RBPs, such as TIA1, HuR, and PABP (30). SGs accumulate upon stress via the formation and fusion of numerous small condensates (31). Importantly, SGs dissolve once stress has passed, allowing SG components to return to performing their soluble function (31, 32). As a subcellular compartment, SGs are central to the pathogenesis of degenerative diseases, as disease-causing mutations in RBPs are associated with accumulation of persistent SGs in ALS, FTD, and IBM (10, 21, 23, 26, 33, 34).

Neurodegenerative diseases are consistently characterized by pathological proteinaceous inclusions, a subset of which contain SG markers (8, 9, 35, 36, 37). A good example is the deposition of TDP-43, which is a prominent feature of approximately 97% of ALS cases, as well as nearly all cases of sporadic IBM and hIBM (10, 24, 35–37). Pathological inclusion formation may be due to the failure of SGs to disassemble (26). It is critical to distinguish whether inclusions observed in postmortem tissues represent persistent SGs or partially disassembled SGs or SG proteins are recruited to preformed inclusions, or some combination of these possibilities (20). Indeed, the precise mechanisms by which specific RBPs become depleted from the nucleus and accumulate in cytoplasmic aggregates remain unclear.

Recent work on the broader group of ventral horn, peripheral nerve, and muscle diseases — including inherited peripheral neuropathy (IPN), hereditary motor neuropathy (HMN), and ALS — has demonstrated a significant genetic overlap, including both pleiotropy and genetic heterogeneity. This overlap is illustrated by findings where causative genes do not exclusively give rise to the phenotype for which they were initially described, as for mutations in *SETX*, *DCTN1*, and *SIGMAR1*, which underlie both ALS and HMN (38–40).

Here, we present patients with 4 potentially novel HNRNPA1 mutations (yielding 3 protein variants: *321Eext*6, *321Qext*6, and G304Nfs*3) and 2 known HNRNPA1 mutations (P288A and D262V). These mutations expand the genetic and clinical spectrum of hnRNPA1-associated neurodegenerative diseases by demonstrating their involvement in complex IPN, atypical ALS, and myopathy. We establish that the mutations can have different effects on hnRNPA1 fibrillization, liquid-liquid phase separation (LLPS), and SG dynamics, indicating the possibility of multiple underlying pathomechanisms for *HNRNPA1* mutations with a possible link to the clinical phenotypes.

## Results

### Identification of HNRNPA1 mutations in 6 separate families

**Figure 1:**
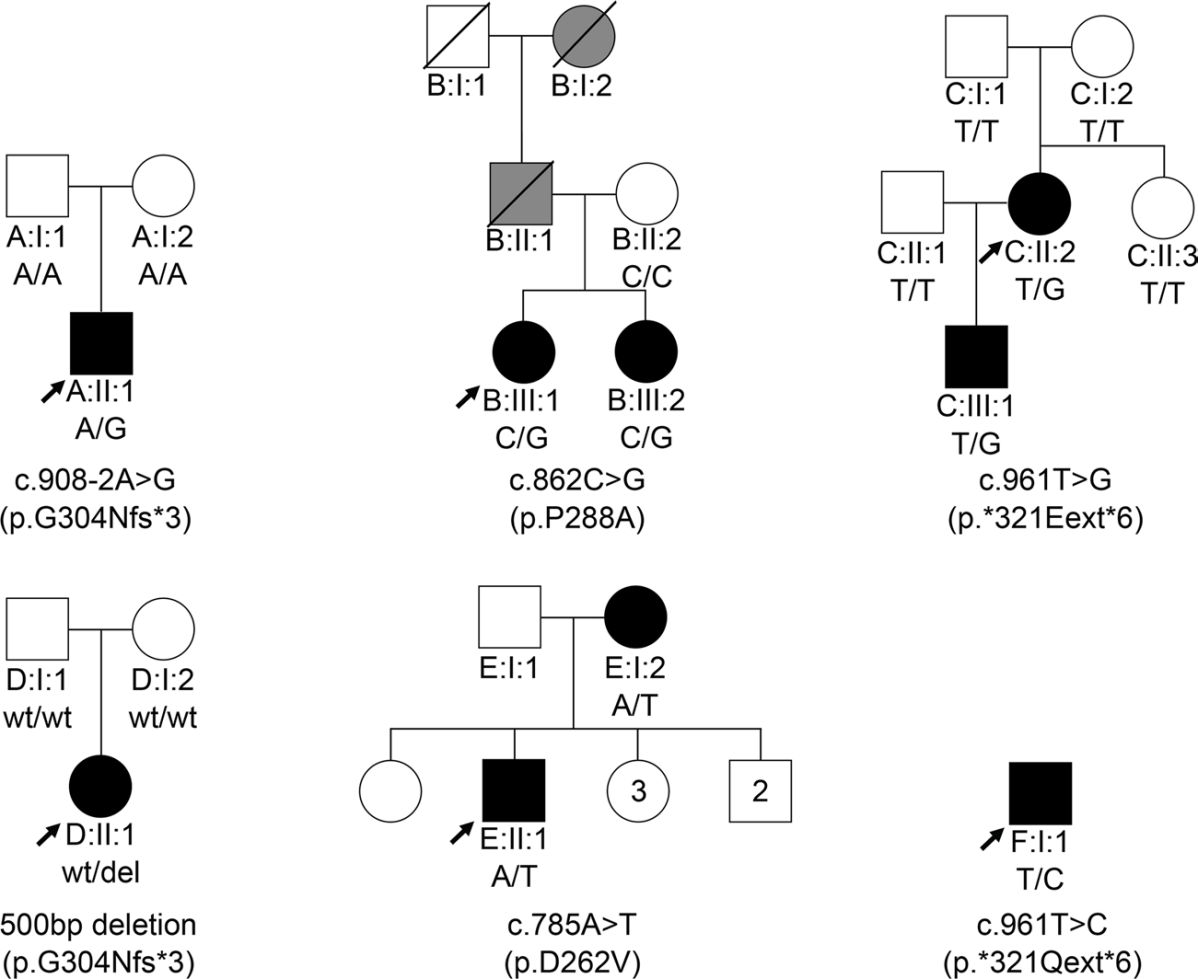
Heterozygous *HNRNPA1* mutations in 6 families. Pedigrees of families A–F with their respective mutations and the segregation of each by genotype, showing affected (black), unaffected (white), and hearsay-affected (gray) individuals. Arrows indicate probands.

**Figure 2:**
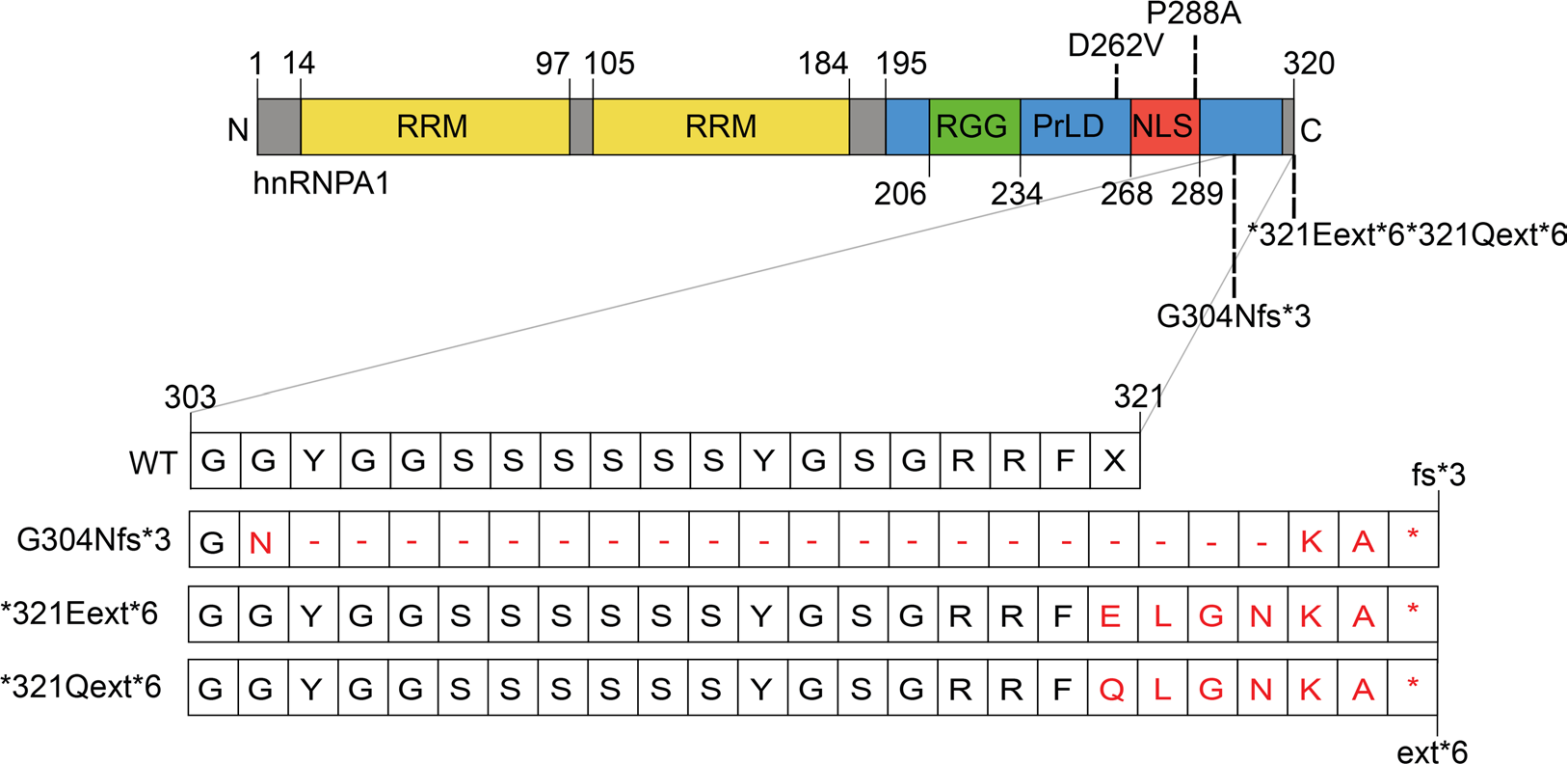
Schematic overview of hnRNPA1 protein and mutations. hnRNPA1 contains 2 N-terminal RNA-recognition motifs (RRMs) and a C-terminal prion-like domain (PrLD), containing an arginine-glycine-glycine (RGG) motif and a proline-tyrosine nuclear localization signal (PY-NLS) that enables nuclear import. Mutations studied in this paper are shown. The effects of the frameshift and extension G304Nfs*3, *321Eext*6, and *321Qext*6 mutations at the protein level are shown in the zoom-in of amino acids 303–321.

Mutations in the *HNRNPA1* gene can result in a range of neurodegenerative disorders (8, 10, 13–15, 17, 41–43). For example, in a patient from family A with motor neuropathy, a heterozygous de novo splice site variation (c.908-2A>G) upstream of the last exon of *HNRNPA1* was identified (Figure 1). cDNA analysis in this patient by PCR and Sanger sequencing revealed skipping of the last exon, producing a transcript that was not degraded by nonsense-mediated decay (NMD), resulting in a truncated protein (p.G304Nfs*3) (Figures 1 and 2 and Supplemental Figure 1; supplemental material available online with this article; https://doi.org/10.1172/jci.insight.148363DS1). To screen for additional hnRNPA1 mutations in patients, we surveyed a large cohort of 547 patients with distal HMN (dHMN), axonal Charcot-Marie-Tooth disease (CMT2), intermediate CMT (CMTi), spinal muscular atrophy (SMA), ALS, hereditary spastic paraplegia (HSP), and myopathy. Thus, we identified a missense variation (c.862C>G; p.P288A) in the index patient of family B that has been linked to ALS (Figure 1) (14). Whole-exome sequencing (WES) for this family did not reveal segregating mutations in other genes known for motor neuron diseases or related disorders. Therefore, the P288A mutation is the most likely cause of disease in family B.

Four additional *HNRNPA1* mutations were identified in families C–F (Figure 1). Whole-genome sequencing (WGS) in family C revealed a single-nucleotide variant in the *HNRNPA1* stop-codon (c.961T>G; p.*321Eext*6) resulting in a stop-loss mutation without undergoing NMD. This mutation added 6 extra amino acids to the protein before ending at an alternative stop-codon (Figure 2). Both this stop-loss mutation and the splice variant from family A terminate on the same TAG sequence present in the WT 3′-UTR (Figure 2). In family D, a 500 bp deletion in *HNRNPA1* was identified through trio WGS (Supplemental Figure 1D). This variant results in the same mRNA alteration as the variant in family A, yielding the same mutant protein (p.G304Nfs*3). For family E, WES in individual E:II:2 identified a known *HNRNPA1* (c.785A>T; p.D262V) missense mutation, which was previously reported in a family with autosomal dominant myopathy and Paget’s disease of bone (PDB) (10, 42). We also identified a nearly identical mutation to *321Eext*6 linked to a similar phenotype in the isolated index of family F (p.*321Qext*6) (Figures 1 and 2 and Supplemental Table 2).

### HNRNPA1 mutations are linked to a variety of phenotypes

#### Family A — (p.G304Nfs*3).

At 15 years of age, the isolated Polish patient (A:II:1) presented the first symptoms with wasting of hand intrinsics. At 16, sural nerve biopsy showed very mild axonal neuropathy with secondary segmental demyelination. Electromyography (EMG) in median, ulnar, and peroneal nerves showed decreased amplitudes of compound muscle action potentials (CMAPs) with nerve conduction velocities in the normal range, while no abnormalities were observed in sensory fibers. At 24, he displayed right-sided thoracic scoliosis, mild pes cavus, wasting of the forearms and calves (lower third), and brisk deep tendon reflexes in the upper and lower limbs with intact sensory modalities. By age 40, he had severe wasting of the distal muscles of the upper and lower limbs. Despite a very clumsy gait with foot drop, he is still ambulant. Overall, his phenotype corresponds to an axonal motor-predominant neuropathy (dHMN).

#### Family B — (p.P288A).

Patient B:II:1 is a 44-year-old woman of Moroccan origin who developed painless proximal right arm weakness and wasting without sensory disturbances at age 22. Her father and paternal grandmother presented the same phenotype. Both died from respiratory failure around the age of 40. She was diagnosed with a very slowly progressive atypical ALS 4 years after symptom onset, based on the clinical features with supportive neurophysiological findings: upper and lower motor neuron symptoms observed at the clinical examination and a chronic neurogenic pattern limited to the upper limb and tongue muscles recorded via EMG. Over time, she developed progressive weakness and wasting of the left arm, respiratory insufficiency, and lower limb spasticity. Five years after symptom onset, she developed bulbar symptoms with dysarthria, rhinolalia, chewing difficulties, and eventually dysphagia for both liquids and solids. She required percutaneous endoscopic gastrostomy 3 years later. After 22 years of disease progression, she is still walking without aid. Her 3 children are unaffected at the ages of 16, 21, and 25. At age 43, her older sister (patient B:II:2) presented with progressive bulbar symptoms, including dysarthria, tongue atrophy, and swallowing problems. She also presented upper motor neuron signs and right hand weakness with amyotrophy. Combined, the phenotype in this family is that of an atypical and slowly progressive ALS.

#### Family C — (p.*321Eext*6).

Patient C:II:2 is of Dutch origin and showed her first symptoms at 9 years of age. She presented with hand function difficulties, mild facial weakness, and general fatigue by the age of 12. Muscle weakness and atrophy were gradually progressive. She has been using a wheelchair since age 34. At age 45, muscle strength in upper and lower limbs had severely decreased, and reflexes were absent in all limbs. She also had a very severe facial weakness and subsequent dysarthria. An quadriceps muscle biopsy was performed, showing nonspecific myopathic changes with some angular fibers but no type grouping. Nerve conduction studies (NCSs) were normal apart from small CMAPs due to severe distal atrophy. Needle EMG was compatible with a myogenic process. The patient developed a mild dissociated vital hyposensitivity distally in the legs, but no abnormalities were noted on spinal MRI. The clinical diagnosis of facio-scapulo-humeral muscular dystrophy could not be confirmed genetically.

The patient’s son (C:III:1) presented his first symptoms with a right-sided foot drop at the age of 8. At 13, he is ambulant and can still walk on his toes. However, heel walking is impossible. He shows slight wasting of the left thenar and reduced muscle strength in the lower limbs. His reflexes are normal in all limbs. NCSs were normal and needle EMG revealed myopathic features. A needle biopsy of the quadriceps muscle was performed at age 8, which showed mild and nonpathognomonic myopathic features. The phenotype in this family is compatible with a myopathy with distal and facial onset and severe proximal and bulbar weakness upon progression. The distal weakness, atrophy, and subsequent small CMAPs suggested an overlap with the dHMN disease spectrum, but myopathic features initially dominated the phenotype.

#### Family D — (p.G304Nfs*3).

Individual D:II:1 is a 35-year-old woman of Asian Indian origin who developed right hand weakness, pain when writing, and atrophy at age 22. At age 26, she was thought to have multifocal motor neuropathy based on her EMG but was treated with a trial of intravenous immunoglobin without response. Her muscle biopsy at age 26 revealed vacuolar myopathy, notable for its myofibrillar disorganization, moth-eaten fibers, rimmed vacuoles, and absence of inflammation. Repeat EMG at age 29 showed irritative distal myopathy. Her symptoms have been gradually progressive: at age 27, she noted right ankle weakness, which is now bilateral. Her exam is notable for mild right facial weakness, and her distal weakness is markedly asymmetric. Reflexes were initially preserved in the lower extremities but have diminished over time; there were no other upper motor neuron signs. She walks unassisted, has scapular winging, and has mild swallowing difficulties with normal speech. She has a restrictive lung function with predicted forced vital capacity of 76% and sleep-disordered breathing. Her creatine kinase has been 2-fold elevated. Cardiac MRI, electrocardiogram, and 14-day cardiac rhythm monitor were normal.

#### Family E — (p.D262V).

Individual E:II:2 is an American male who developed asymmetric weakness of the right foot at around 36 years of age, worsening over time to involve bilateral proximal leg weakness. He initially noticed difficulties with snow skiing and later had frequent tripping and falls. Over the course of 10 years, there was slow progression of bilateral leg weakness that now requires use of a cane for ambulation. Currently, at age 48, the patient uses his arms to stand from sitting position and requires handrails when going up the stairs. He reports no weakness of facial, neck, arm, or trunk muscles; numbness or tingling; dysphagia; difficulty with handwriting, handling utensils, or opening doors; shortness of breath; bowel/bladder dysfunction; diplopia; or muscle pain. NCS abnormalities were limited to low amplitude of the right fibular CMAPs, whereas needle EMG revealed chronic myopathic changes proximally and distally in the leg. A muscle biopsy at age 45 showed chronic myopathy with rimmed vacuoles.

The patient’s mother (E:I:2) was diagnosed with PDB at the age of 48. Within 3 years she developed left leg weakness that gradually progressed to involve both legs. She is currently using a wheelchair. She has normal arm strength and normal cranial nerve function, and a muscle biopsy was consistent with myopathy with inclusion bodies. The index patient has 6 unaffected siblings (34–50 years old). The overall phenotype in this family is consistent with inclusion body myositis.

#### Family F — (p.*321Qext*6).

Patient F:I:1 is of Belgian origin and presented first at the age of 12 years with muscle atrophy in the hands, with subsequent progressive wasting of the lower limbs starting distally before also involving proximal muscles. At his last examination at the age of 64, this patient used a wheelchair with profound generalized weakness resulting in de facto quadriplegia. Reflexes were lost in all extremities. In addition to contractures in the lower limbs and bilateral *pes equinovarus*, the patient displayed marked facial weakness. Respiration was severely limited, requiring cough assist and noninvasive home ventilation (bilevel positive airway pressure). The patient also made use of a percutaneous endoscopic gastrostomy. A biopsy of the tibialis anterior muscle at the age of 13 years showed myogenic alterations consisting of marked diameter variation, selective type 1 fiber atrophy, increased centralized nuclei, and the presence of rimmed vacuoles. A biopsy of the fibularis superficialis nerve was strictly normal. NCSs and needle EMG did not show neurogenic changes and were consistent with a distally predominant myopathy. The patient died at age 64. The family history for this patient is negative for neuromuscular disorders, making this an isolated case. The clinical presentation fits with a progressive myopathy with onset in the distal limbs. A clinical diagnosis of distal myopathy of Welander type or Nonaka type was suggested. No mutation in UDP-*N*-acetylglucosamine-2-epimerase/*N*-acetylmannosamine kinase (*GNE*) was found.

### Comparable in vitro transcription and translation of hnRNPA1 mutants

To verify that the alternatively terminating variants were not subject to NMD, mRNA sequences for the G304Nfs*3 (family A) and *321Eext*6 (family C) variants were assessed by PCR amplification from exon 8 into the 3′-UTR on cDNA from patient lymphoblasts. Gel electrophoresis and di-deoxy sequencing confirmed exon 11 skipping for the G304Nfs*3 splice variant (Supplemental Figure 1A). mRNA for the G304Nfs*3 and *321Eext*6 variants was present, indicating the absence of NMD and suggesting that these genes are transcribed and likely translated in patients (Supplemental Figure 1, B and C). RNA sequencing confirmed altered splicing in family D, resulting in the same protein as family A (Supplemental Figure 1D). Next, hnRNPA1 plasmids were constructed for N-terminally V5 tagged WT hnRNPA1 cDNA, as well as P288A, G304Nfs*3, and *321Eext*6 mutations, and each protein was transiently expressed in Henrietta Lacks (HeLa) cells. Immunoblotting confirmed translation of WT hnRNPA1 and the mutant proteins. As expected, the G304Nfs*3 mutation produced a shorter protein, whereas the *321Eext*6 mutation yielded a longer protein (Supplemental Figure 1E). The P288A mutation translated into a protein of similar size to WT hnRNPA1 (Supplemental Figure 1E). Thus, these patient-derived mutant mRNA transcripts can be translated into mutant hnRNPA1 protein in cells.

### WT and mutant hnRNPA1 confer toxicity in yeast

Next, we assessed the hnRNPA1 variants in a yeast model of hnRNPA1 aggregation and toxicity (10). The hnRNPA1 PY-NLS is not decoded by the yeast nuclear import machinery, and hnRNPA1 localizes to the cytoplasm in yeast (44–46). hnRNPA1 and hnRNPA1^D262V^ form cytoplasmic aggregates in yeast and are toxic, which phenocopies pathological events in MSP (10, 47). Here, it is important to note that in MSP cases caused by mutations in *VCP* or hnRNPA2, it is the WT version of hnRNPA1 that undergoes cytoplasmic aggregation (10). Thus, expression of WT hnRNPA1 in yeast recapitulates the cytoplasmic mislocalization and aggregation of WT hnRNPA1 that occurs in MSP caused by mutations in hnRNPA2 or VCP (10).

**Figure 3:**
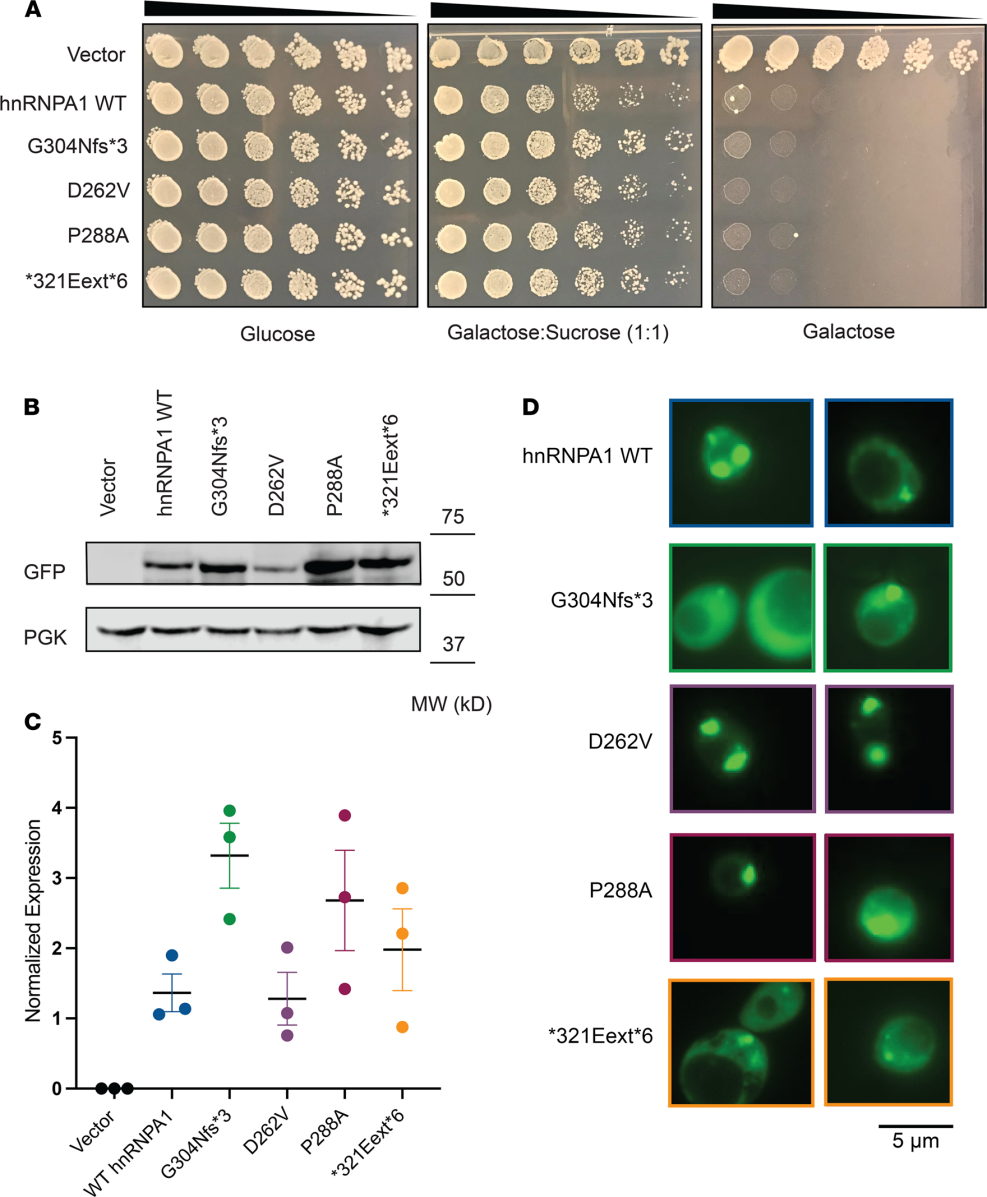
hnRNPA1 variants are toxic and aggregate in yeast. (**A**) GFP-tagged hnRNPA1 variants are toxic when expressed in yeast. Yeast spotting assays compare the toxicity of hnRNPA1 variants by plating yeast at serially diluted concentrations. hnRNPA1 variants were expressed from a galactose-inducible promoter for 72 hours. Expression of hnRNPA1 is repressed when cells are grown on glucose (left panel), then moderately induced on sucrose/galactose (middle panel), and strongly induced on galactose (right panel). (**B**) Western blot confirms the expression of GFP-tagged hnRNPA1 variants in yeast after 8 hours in galactose. Phosphoglycerate kinase (PGK) is used as loading control. (**C**) GFP signal was quantified from 3 separate blots and normalized to the PGK signal of the respective sample. The variability in hnRNPA1 expression is not significant as measured by 1-way ANOVA using Dunnett’s multiple comparisons test. (**D**) Yeast cells expressing GFP-tagged WT and mutant hnRNPA1. hnRNPA1, hnRNPA1^D262V^, hnRNPA1^P288A^, and hnRNPA1^*321Eext*6^ formed cytoplasmic foci. hnRNPA1^G304Nfs*3^ displayed more diffuse localization in yeast, although cytoplasmic foci could be found.

We transformed WT or mutant hnRNPA1 on a galactose-induced expression vector and serially diluted yeast onto glucose (no expression of hnRNPA1), sucrose/galactose (1:1; moderate expression of hnRNPA1), or galactose-containing agar plates (high expression of hnRNPA1). Expression of WT or mutant hnRNPA1 on sucrose/galactose (1:1) and galactose media was toxic (Figure 3A). Immunoblot confirmed that hnRNPA1 variants were robustly expressed in yeast on galactose media (Figure 3, B and C). Thus, in yeast, the hnRNPA1 variants displayed similar toxicity to WT hnRNPA1, suggesting that the cytoplasmic localization of hnRNPA1 is pathogenic for these variants in yeast (Figure 3A).

Next, we assessed the aggregation of mutant hnRNPA1. hnRNPA1, hnRNPA1^D262V^, hnRNPA1^P288A^, and hnRNPA1^*321Eext*6^ formed cytoplasmic foci (Figure 3D). By contrast, hnRNPA1^G304Nfs*3^ displayed more diffuse localization, although cytoplasmic foci were still evident (Figure 3D). G304Nfs*3 was expressed to similar levels as the other hnRNPA1 proteins (Figure 3, B and C). Thus, hnRNPA1^G304Nfs*3^ may be less aggregation prone than hnRNPA1, hnRNPA1^D262V^, hnRNPA1^P288A^, and hnRNPA1^*321Eext*6^ while conferring similar toxicity (Figure 3A).

### Differential fibrillization and LLPS propensity of hnRNPA1 variants

**Figure 4:**
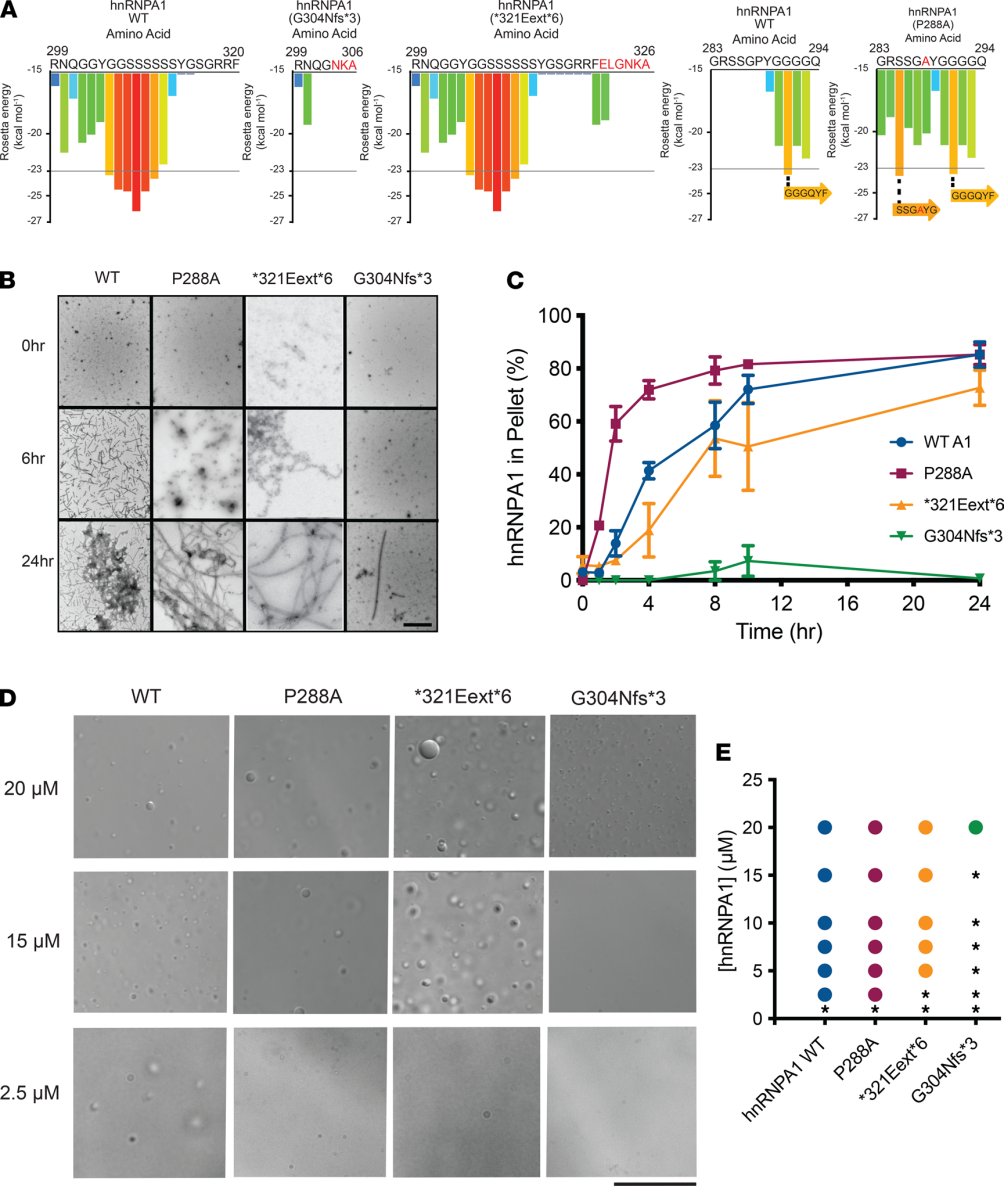
hnRNPA1 variants can exhibit altered propensity for fibrillization and LLPS. (**A**) ZipperDB calculates the propensity of hexapeptide fragments to form steric zippers (48). Steric zippers, which are self-complementary β-sheets that form the backbone of amyloid fibrils, are predicted to form when the Rosetta energy of a hexapeptide is below the empirically determined “high fibrillization propensity” threshold of −23 kcal/mol (48). P288A introduces a potent steric zipper (285-SSGAYG-290) that could increase the fibrillization propensity. G304Nfs*3 deletes several potent steric zippers, which could reduce fibrillization propensity. (**B** and **C**) GST-TEV-hnRNPA1 and disease variants (5 μM) were incubated with TEV protease in A1 assembly buffer to initiate fibrillization. Reactions were agitated at 1200 rpm or 0–24 hours at 25°C. Fibrillization was monitored by electron microscopy (**B**). Scale bar, 0.5 μm. Alternatively, hnRNPA1 fibrillization kinetics were determined by sedimentation analysis (**C**) where the amount of hnRNPA1 in the pellet fraction was quantified. Values represent average ± SEM (*n* = 3–6). (**D**) Representative differential interference contrast (DIC) microscopy images of hnRNPA1 droplets formed by different hnRNPA1 variants. Droplets were formed by combining the indicated hnRNPA1 variant at the indicated concentration in a LLPS buffer and were imaged immediately after all components had been added. Scale bar: 25 μm. (**E**) Phase diagram of hnRNPA1 variants showing the hnRNPA1 concentrations where LLPS occurs. hnRNPA1 and hnRNPA1^P288A^ form droplets at concentrations of 2.5 μM or higher. hnRNPA1^*321Eext*6^ forms droplets at concentrations of 5 μM or higher. hnRNPA1^G304Nfs*3^ forms droplets at concentrations of 20 μM or higher. At each concentration, a colored circle indicates droplet formation, whereas a black asterisk indicates no droplets.

Our yeast work suggested that hnRNPA1 variants may differ in their propensity to self-assemble. To investigate how the mutations affect the fibrillization propensity of hnRNPA1, we first examined WT hnRNPA1 and the disease-associated mutants with ZipperDB (48). ZipperDB is a structure-based threading algorithm, which scores 6 amino acid segments for their propensity to form 2 self-complementary β-strands termed “steric zippers” that form the spine of amyloid fibrils. Hexapeptides with Rosetta energy of lower than –23 kcal/mol are predicted to form steric zippers, with lower energy predicting higher amyloidogenicity. ZipperDB predicted that the P288A mutation introduces a new steric zipper (285-SSGAYG-290) that could increase the fibrillization propensity (Figure 4A). By contrast, G304Nfs*3 deletes a large stretch of steric zippers present in the C-terminal portion of the PrLD, which could reduce multivalency and aggregation propensity (Figure 4A). Finally, the *321Eext*6 mutation did not change the steric zipper landscape, with ZipperDB predicting a similar fibrillization propensity to WT hnRNPA1 (Figure 4A).

To assess the fibrillization propensity, we purified GST-TEV–tagged versions of the proteins from *E*. *coli* (10). Fibrillization of purified GST-TEV-hnRNPA1 was initiated by incubating with TEV protease to remove the GST tag (10). Fibrillization kinetics were then monitored by sedimentation analysis, and the final structures that formed were imaged via electron microscopy (10). hnRNPA1^P288A^ assembled into fibrils more rapidly than WT hnRNPA1 (Figure 4, B and C), consistent with the ZipperDB prediction (Figure 4A). Thus, P288 disfavored β-sheet interactions between hnRNPA1 proteins that drive fibrillization (Figure 4A) (49–51). P288 is also a critical component of the hnRNPA1 PY-NLS, which is important for nuclear localization (52, 53). Therefore, in cells, the P288A mutation contributes to increased cytoplasmic hnRNPA1 mislocalization and aggregation. As predicted by ZipperDB, hnRNPA1^*321Eext*6^ exhibited similar fibrillization kinetics to WT hnRNPA1 (Figure 4, B and C). Strikingly, hnRNPA1^G304Nfs*3^ displayed decelerated fibrillization, with only a few fibrils apparent after 24 hours (Figure 4, B and C), again in line with ZipperDB predictions (Figure 4A) and reduced formation of cytoplasmic foci in yeast (Figure 3D). Thus, P288A accelerates hnRNPA1 fibrillization, whereas *321Eext*6 has minimal effect, and G304Nfs*3 reduces fibrillization.

hnRNPA1 is a component of SGs, which form via LLPS (10, 25). Similar to other PrLD-containing RBPs in SGs, purified hnRNPA1 forms liquid droplets spontaneously upon macromolecular crowding (25). In the presence of a crowding agent, WT hnRNPA1 forms liquid droplets at concentrations of 2.5 μM or greater (Figure 4, D and E). hnRNPA1^P288A^ showed similar LLPS properties to WT hnRNPA1 (Figure 4, D and E), whereas hnRNPA1^*321Eext*6^ had slightly reduced ability to undergo LLPS, as no droplets formed at 2.5 μM (Figure 4, D and E). Thus, the features that drive fibrillization of hnRNPA1 (such as steric zippers) do not necessarily have the same effect on LLPS (54). By contrast, hnRNPA1^G304Nfs*3^ exhibited reduced LLPS as liquid droplets were only observed at 20 μM (Figure 4, D and E). Moreover, hnRNPA1^G304Nfs*3^ droplets were smaller than those formed by hnRNPA1, hnRNPA1^P288A^, and hnRNPA1^*321Eext*6^ (Figure 4, D and E). Thus, the G304Nfs*3 mutation perturbs hnRNPA1 LLPS and fibrillization, likely by reducing PrLD multivalency (Figure 4A).

### hnRNPA1 mutants modulate SG dynamics

**Figure 5:**
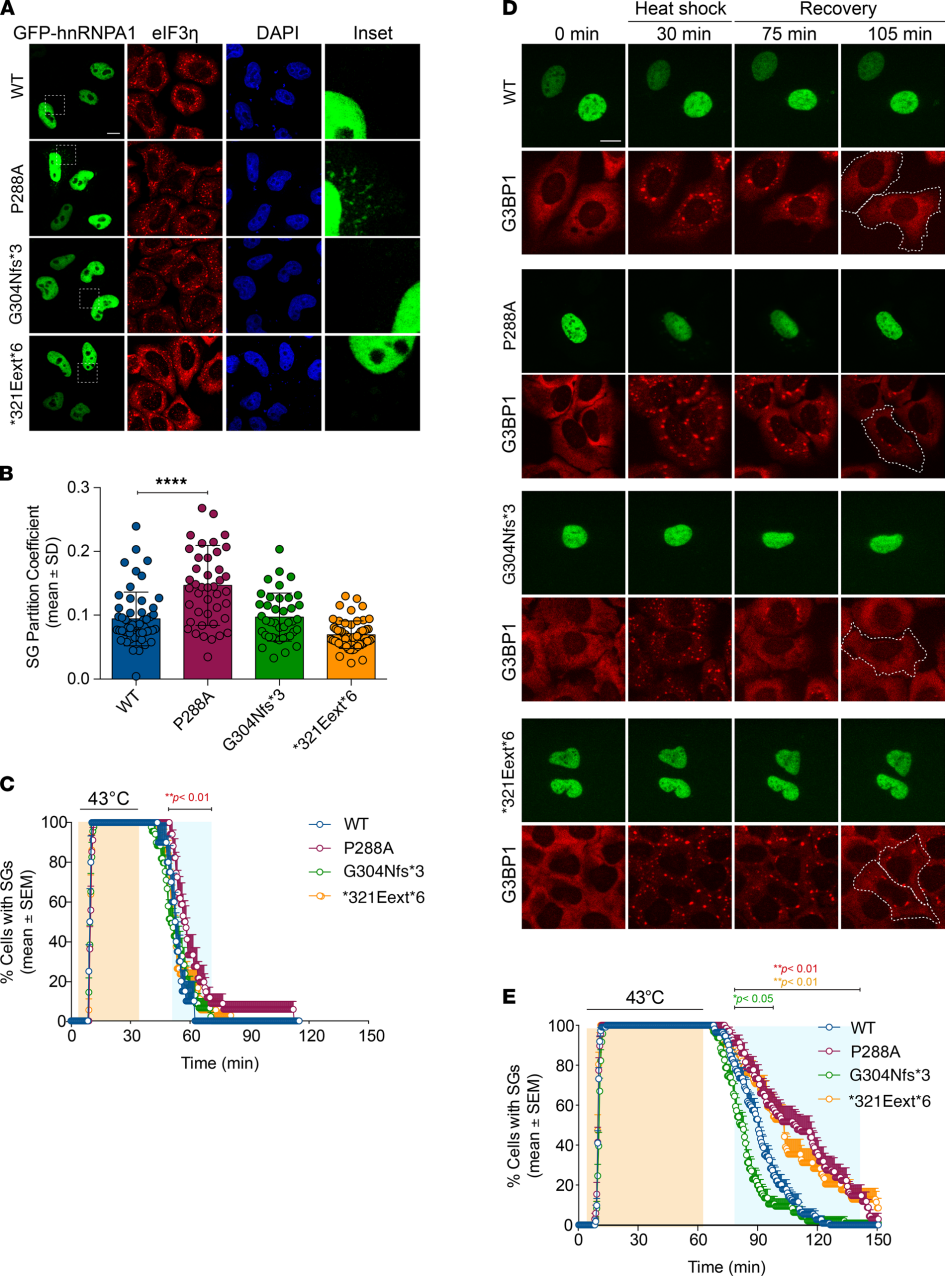
Localization and stress granule dynamics of the hnRNPA1 mutants. (**A** and **B**) HeLa cells were transiently transfected with WT or mutant EGFP-tagged hnRNPA1 and subjected to arsenite stress (0.5 mM sodium arsenite, 30 minutes). Cells were fixed and stained with eIF3η (red) and DAPI (blue). Confocal images were taken for partition coefficient analysis. Scale bar: 10 μm (original magnification, ×10 for the insets). Error bars represent mean ± SD (*n* = 55, 43, 40, and 58 cells for WT, P288A, G304Nfs*3, and *321Eext*6, respectively). ****P < 0.0001, by ordinary 1-way ANOVA with Dunnett’s multiple comparisons test. (**C**) U2OS cells expressing tdTomato-tagged endogenous G3BP1 were transiently transfected with WT or mutant EGFP-tagged hnRNPA1 and subjected to heat shock (43°C, 30 minutes; orange shading) and allowed to recover at 37°C for 2 hours. Line graph represents the percentage of cells with visible tdTomato-G3BP1 puncta over time. Error bars represent mean ± SEM (*n* = 10, 17, 23, and 18 videos for WT, P288A, G304Nfs*3, and *321Eext*6, respectively). Blue shaded area indicates time points at which P288A mutant was statistically significantly different from WT. **P < 0.01 by 2-way ANOVA with Dunnett’s multiple comparisons test. (**D** and **E**) U2OS cells expressing tdTomato-tagged endogenous G3BP1 were transiently transfected as in **C** and subjected to heat shock (43°C, 60 minutes; orange shading) and allowed to recover at 37°C for 90 minutes. White dotted lines (**D**) delineate hnRNPA1-positive cells; scale bar indicates 10 μm. Line graph (**E**) represents the percentage of cells with visible tdTomato-G3BP1 puncta over time (*n* = 56, 34, 48, and 35 videos for WT, P288A, G304Nfs*3, and *321Eext*6, respectively). Blue shaded area indicates time points at which each mutant was statistically significantly different from WT. *P < 0.05, **P < 0.01 (colors correspond to the respective mutants), by 2-way ANOVA with Dunnett’s multiple comparisons test.

In human cells, hnRNPA1 localizes to SGs in response to specific stressors (10, 55, 56). Thus, we expressed WT or mutant GFP-tagged hnRNPA1 in HeLa cells and subjected these cells to environmental stressors. WT hnRNPA1 was predominantly nuclear upon arsenite stress (Figure 5, A and B). G304Nfs*3 and *321Eext*6 mutations did not affect incorporation of hnRNPA1 into arsenite-induced SGs, whereas hnRNPA1^P288A^ showed significantly greater incorporation into SGs (Figure 5, A and B). This result is consistent with previous reports that mutations in the hnRNPA1 PY-NLS reduce hnRNPA1 nuclear import and enhance incorporation into SGs (13, 14, 57). With heat stress, we observed greater WT hnRNPA1 incorporation into SGs (Supplemental Figure 2). The *321Eext*6 mutation did not affect hnRNPA1 incorporation into heat shock–induced SGs, whereas hnRNPA1^P288A^ showed significantly greater incorporation (Supplemental Figure 2). G304Nfs*3 reduced incorporation of hnRNPA1 into SGs upon heat shock, suggesting that P288A and G304Nfs*3 mutations have opposite effects on hnRNPA1 incorporation into SGs.

To assess how hnRNPA1 mutations affect SG dynamics, we established a live-cell assay that monitors SG assembly and disassembly kinetics during a controlled heat pulse. Thus, we generated a U2OS cell line in which CRISPR/Cas9 was used to tag endogenous G3BP1 (a SG marker) with tdTomato (58). We transiently transfected these cells with EGFP-tagged hnRNPA1 (WT or mutant forms). Although none of the hnRNPA1 mutants had a significant effect on the number or assembly rate of SGs, we observed mutation-dependent effects on SG disassembly kinetics. When SGs were induced by a 30-minute heat shock, the hnRNPA1^P288A^ slightly delayed disassembly relative to WT hnRNPA1 (Figure 5C). Extending the heat shock to 60 minutes further delayed SG disassembly for hnRNPA1^P288A^, and now hnRNPA1^*321Eext*6^ also decelerated SG disassembly (Figure 5, D and E). Cells expressing WT hnRNPA1 or hnRNPA1^G304Nfs*3^ showed nearly complete SG disassembly after 60-minute recovery (Figure 5, D and E). Consistent with our pure protein data (Figure 4D), hnRNPA1^G304Nfs*3^ elicited faster SG disassembly than WT hnRNPA1 (Figure 5, D and E). However, approximately 50% of cells expressing hnRNPA1^P288A^ or hnRNPA1^*321Eext*6^ failed to disassemble SGs in this time frame (Figure 5, D and E). This delay suggests that incorporation of hnRNPA1^P288A^ or hnRNPA1^*321Eext*6^ into SGs alters their dissolution. Moreover, these findings suggest that the severity of stress can reveal differences between hnRNPA1 variants.

Finally, we evaluated how hnRNPA1^D262V^, which is associated with MSP and was identified in family E in this study, altered SG formation (10). As with the other mutations tested here, hnRNPA1 incorporation into SGs was similar for WT hnRNPA1 and hnRNPA1^D262V^ (Supplemental Figure 3A). However, similar to hnRNPA1^P288A^ or hnRNPA1^*321Eext*6^, hnRNPA1^D262V^ significantly delayed SG disassembly (Supplemental Figure 3, B and C). Thus, hnRNPA1 mutations can have variable effects on SG disassembly kinetics. Specifically, P288A, D262V, and *321Eext*6 can delay SG disassembly, whereas G304Nfs*3 can accelerate SG disassembly.

## Discussion

Here, we identified and characterized potentially novel and previously described *HNRNPA1* mutations in patients with atypical ALS, HMN, distal myopathy, and MSP3 (Supplemental Table 2). In 6 families, we identified stop-loss mutations *321Eext*6 and *321Qext*6 leading to extension of the protein; a splice site mutation and a 500 bp deletion, both leading to skipping of the last exon, resulting in a truncated protein, G304Nfs*3; a known missense mutation causal for ALS (P288A) in a family with an atypical slowly progressive ALS phenotype; and the known MSP3 D262V variant in a family with myopathy (14). These mutations are all in the hnRNPA1 PrLD, supporting previous findings that PrLD alterations can have important biological consequences (10, 50). Despite the larger cohort that was surveyed, we only identified 4 *HNRNPA1* mutations, suggesting that *HNRNPA1* mutations are rare.

Mutations in *HNRNPA1*, though rare, show great clinical heterogeneity. Patients in family A (G304Nfs*3) and B (P288A) present with phenotypes of pure motor neuron disorders without sensory involvement and with pyramidal, bulbar, and upper motor neuron involvement spanning the spectrum of dHMN to atypical (juvenile) ALS. The slow progression of disease in these patients is striking. Patients in family B remain ambulant even 15–20 years after disease onset, unlike typical ALS cases. Patients in family C (*321Eext*6) present a phenotype that is compatible with a distal-onset myopathy with facial weakness with relatively faster progression, with the eldest patient using a wheelchair by age 34. Some neurogenic features suggest an overlap with the motor neuron spectrum in this family. Family F (*321Qext*6) shows a myopathic phenotype with pronounced facial weakness similar to family C. These families harbor the similar mutations *321Eext*6 (family C) and *321Qext*6 (family F). The myopathic phenotype in family E (D262V) is similar to the previously reported family carrying the D262V mutation. These families demonstrate that a broader range of phenotypes is associated with pathogenic *HNRNPA1* variants than was previously appreciated. Based on these findings, we suggest that it could be beneficial to test patients with a range of motor neuron diseases, including dHMN, atypical and typical ALS, as well as (distal) myopathy and MSP phenotypes, for pathogenic *HNRNPA1* variants. Defining the mechanism by which hnRNPA1 mutations manifest pathologically is essential for understanding and treating diseases in which these mutations are present.

Several RBPs with PrLDs, including hnRNPA1, are implicated in neurodegenerative diseases (8, 10, 35, 47, 59–63). Disease-linked PrLD mutations can exacerbate the propensity of the RBP to form self-seeding fibrils, resulting in cytoplasmic aggregation and persistent SGs (8, 10, 23, 41). In addition to negative gain-of-function consequences of PrLD mutations, there are also loss-of-function consequences to having a mutated PrLD. In the case of hnRNPA1, the PrLD participates in pre-mRNA splicing, stable RNA binding, optimal RNA annealing activity, and protein-protein interactions (64, 65). Thus, PrLD mutations may also perturb normal hnRNPA1 function.

We assessed the intrinsic fibrillization and LLPS propensity of 4 of the *HNRNPA1* mutations identified here (Table 1). P288A is predicted to accelerate fibrillization because proline can act as a gatekeeper residue that inhibits fibrillization by inducing a local twist (structural constraint) that is incompatible with cross-β structure (49–51). ZipperDB predicted that the P288A mutation results in a new potent steric zipper in the PrLD, which we show causes more rapid fibrillization. This change in the biophysical properties of hnRNPA1^P288A^ is compounded by its impaired nuclear import (13). Indeed, the P288A mutation alters the critical proline of the PY-NLS of hnRNPA1, which weakens the interaction with its nuclear import receptor, karyopherin-β2 (Kapβ2) (53, 66). Thus, Kapβ2 is predicted to be less able to chaperone and disaggregate hnRNPA1^P288A^ in the cytoplasm and return hnRNPA1^P288A^ to the nucleus (66). Thus, enhanced intrinsic fibrillization and reduced interaction with Kapβ2 likely combine to render P288A pathogenic. In this way, the hnRNPA1^P288A^ variant resembles ALS-linked FUS^P525L^ where the critical proline of the FUS PY-NLS is mutated to leucine, which reduces the interaction with Kapβ2 (66, 67) and simultaneously directly promotes FUS aggregation (68).

ZipperDB predicted that the *321Eext*6 mutation does not alter the steric zipper landscape of hnRNPA1. Accordingly, hnRNPA1^*321Eext*6^ exhibited similar fibrillization propensity as WT hnRNPA1. Finally, ZipperDB revealed that the G304Nfs*3 mutation deletes several potent steric zippers from the PrLD, which would reduce the multivalency of the PrLD and reduce fibrillization. Indeed, hnRNPA1^G304Nfs*3^ exhibited reduced fibrillization in vitro. The decelerated fibrillization of G304Nfs*3 is also observed in other mutant proteins connected with neurodegenerative disease. For example, the α-synuclein variant, A30P, which causes familial Parkinson’s disease (69), assembles into fibrils more slowly than WT α-synuclein (70). However, α-synuclein^A30P^ accesses toxic oligomeric forms more rapidly than WT α-synuclein and populates these forms for longer before forming fibrils (70). A similar situation could arise with G304Nfs*3, with the extended lag phase of fibrillization causing prolonged exposure to toxic oligomeric species.

In addition to forming stable fibrils, the hnRNPA1 PrLD promotes LLPS, which contributes to SG assembly (25, 54). hnRNPA1^G304Nfs*3^ exhibited reduced LLPS propensity, whereas hnRNPA1^P288A^ and hnRNPA1^*321Eext*6^ were more similar to WT hnRNPA1. The reduced LLPS propensity of hnRNPA1^G304Nfs*3^ could lead to loss of hnRNPA1 function for processes that require hnRNPA1 LLPS, such as (but not limited to) maintaining SG stability (71–74).

Upon stress, hnRNPA1 partitions into cytoplasmic SGs, only to disperse again once stress dissipates. *HNRNPA1* mutations also affect SG dynamics. Fibrillization-prone hnRNPA1^P288A^ increased hnRNPA1 accumulation in SGs and decelerated SG disassembly. Conversely, hnRNPA1^G304Nfs*3^, which shows impaired LLPS and fibrillization, facilitated more rapid SG disassembly. hnRNPA1^*321Eext*6^ exhibited similar fibrillization propensity to WT hnRNPA1 but delayed SG disassembly. hnRNPA1^*321Eext*6^ might slow SG disassembly via the C-terminal -ELGNKA extension, which may enable SG-stabilizing interactions.

Intracellular RBP aggregates are a hallmark of ALS/FTD. However, different hypotheses have been advanced to explain how mutations in these proteins cause neurodegeneration. On the one hand, pathological aggregates can cause a toxic gain of function (75). On the other hand, mutations can lead to loss of normal function (75). Loss of hnRNPA1 function could affect splicing, translation, miRNA biogenesis, and transcription of many targets.

Our findings suggest that disease-linked *HNRNPA1* mutations can have different effects on fibrillization, LLPS, and SG dynamics while still causing phenotypes within the known spectrum of disease. Thus, rather than a universal mechanism defined by accelerated fibrillization as with D262V, P288A, and D262N (10), it seems likely that there are multiple disease-causing mechanisms at work as suggested by the *321Eext*6 and G304Nfs*3 variants. Which critical hnRNPA1 functions are most affected and how they affect physiological pathways remains to be investigated. Further research into the differences in normal functions affected by the mutations might help explain the phenotypic variation observed for *HNRNPA1* mutations. The identification of additional mutations in hnRNPA1 in degenerative disorders of motor neurons and muscle will also help understand the underlying pathomechanisms. Based on our results, we suggest that there is not one unifying mechanism but rather a spectrum of disturbances, including fibrillization propensity, LLPS propensity, and SG dynamics, each of which may elicit cellular dysfunction and degeneration.

## Methods

### Study participants.

We describe 6 families: 3 with isolated patients and 3 with a dominant inheritance of motor neuron or muscle disorders (Supplemental Table 2). Samples from these 6 families were subjected to next-generation sequencing. Additionally, 547 patients diagnosed with dHMN, CMT2, CMTi, SMA, ALS, HSP, or myopathy were screened for *HNRNPA1* mutations. For all patients and family members, high-molecular-weight genomic DNA was extracted from blood samples using standard methods.

### Next-generation sequencing.

Next-generation sequencing (WES or WGS) was performed on high-molecular-weight genomic DNA from families A–F (Supplemental Table 1). Subsequent processing and filtering were performed in the separate research or diagnostic facilities using similar approaches and general practices used for identification of rare disease-causing genetic variants (Supplemental Table 1).

The de novo heterozygous 500 bp deletion identified in family D encompassing exon 9 of *HNRNPA1* (NM_002136.4: c.907+15_*5-68del) (76, 77) was confirmed via a quantitative PCR assay. Transcriptome sequencing (RNA-Seq) was performed on D:II:1 cultured fibroblasts to assess the effect of the deletion on the *HNRNPA1* mRNA transcript (78). RNA-Seq revealed skipping of exon 9 (https://varsome.com/transcript/hg19/NM_002136.4), which is predicted to result in a truncated protein (p.G304Nfs*3) (Figure 2 and Supplemental Figure 1D). Additionally, RNA-Seq did not show a decrease in *HNRNPA1* mRNA expression levels compared with controls, indicating the aberrant transcript escapes NMD (Supplemental Figure 1D).

Requests for deidentified sequencing data sets for the families included in this study should be directed to the corresponding author, JB.

### HNRNPA1 variant screening.

A Multiplex Amplification of Specific Targets for Resequencing (Agilent) assay was designed to screen for mutations in all coding regions and exon-intron boundaries of the *HNRNPA1* gene in an additional 547 patients. Multiplex PCRs and following barcoding steps were performed on a Veriti AB machine (Life Technologies). Libraries were sequenced on a MiSeq Platform (Illumina) using v3 chemistry with read lengths of 2 × 300 bp. For read alignment, variant calling, annotation, and filtering, GATK, SAMtools, and GenomeComb were used. For possible pathogenic *HNRNPA1* variants, WES was performed to exclude other pathogenic variants, and Sanger sequencing was used for validation and segregation analysis in all available family members.

### Plasmid generation.

mRNA for index patient family A, index patient family C, and a control individual was extracted from lymphoblast cell lines by RNeasy Mini Kit (QIAGEN). cDNA was generated by reverse transcription using the SuperScript III First-Strand Synthesis System (Thermo Fisher Scientific). cDNA was used for PCR amplification of *HNRNPA1* (NM_002136.3) with primers containing sequences suitable for subsequent Gateway Cloning Technology (Thermo Fisher Scientific). pDONR221 was used as the entry vector. The P288A variant from family B was introduced into the WT *HNRNPA1* entry clone by site-directed mutagenesis (79). The G304Nfs*3 mutation from families A and D and the *321Eext*6 variant from family C were cloned by direct amplification of the patient cDNA. The mRNA for the variant from family D is the same as the variant for family A, and thus the same plasmid was used. The entry clones were subcloned into a pLenti backbone with N-terminal V5- or EmGFP-tag for stable transduction and puromycin selection. Templates were checked by Sanger sequencing.

Yeast plasmids with each hnRNPA1 variant were generated by cloning into the pAG416GAL-GFP-ccdB vector backbone using Gateway reactions (Thermo Fisher Scientific). Mutations were generated via QuikChange Site-Directed Mutagenesis Kit (Agilent) and verified by DNA sequencing. Plasmids for bacterial protein expression were generated by cloning into a pDUET-GST vector backbone. Mutations were made via QuikChange Site-Directed Mutagenesis Kit (Agilent) and verified by DNA sequencing.

### Mammalian cell culture.

HeLa (CCL-2) and U2OS (HTB-96) cells were from ATCC; cultured in DMEM (HyClone) supplemented with 10% fetal bovine serum (FBS; HyClone; SH30396.03), 1× GlutaMAX (Thermo Fisher Scientific; 35050061), and 50 U/mL penicillin and 50 μg/mL streptomycin (Gibco; 15140–122); and maintained at 37°C in a humidified incubator with 5% CO_2_. U2OS cells expressing tdTomato-tagged endogenous G3BP1 have been described (58). Cells were authenticated by short tandem repeat profiling.

### Western blot.

HeLa cells were incubated at 37°C in a humidified atmosphere with 5% CO_2_. Cells were cultured in MEM, with l-glutamine and Earle’s salts (Thermo Fisher Scientific), with 10% heat-inactivated FBS, 1% l-glutamine, and 1% penicillin-streptomycin. Transient transfection of HeLa cells with WT and mutant HNRNPA1 lentiviral plasmids was performed using polyethylenimine. Transfected HeLa cells were cultured for 24 hours before collection.

After transfection, HeLa cells (4 *×* 10^6^) were pelleted and lysed with RIPA lysis buffer (150 mM NaCl, 0.5% sodium deoxycholate, 0.1% sodium dodecyl sulfate [SDS], 1% NP-40, 50 mM Tris-HCl pH 7.4) supplemented with protease inhibitors (Roche Diagnostics). A total of 12.5 μg protein was run on a NuPAGE Novex 4%–12% Bis-Tris Protein Gel (Thermo Fisher Scientific). Primary antibody mouse anti-V5 (1:5000; Life Technologies R960-25) and secondary goat anti–mouse IgG2a horseradish peroxidase (1:10,000; Southern Biotech, catalog 1081-05) were used, and visualization was performed with enhanced chemiluminescence detection (Pierce ECL Plus Western Blotting Substrate, Thermo Fisher Scientific). Equal loading was assessed by rabbit α-tubulin primary antibody (1:5000; Abcam rabbit polyclonal IgG ab4074) with goat anti–rabbit IgG horseradish peroxidase secondary antibody (1:10,000; Southern Biotech, catalog 4030-05).

Yeast cells transformed with the appropriate plasmid were grown at 30°C in galactose-containing media for 8 hours to induce protein expression. Cultures were normalized to an OD_600_ = 0.6, and 6 mL of cells were harvested. Yeast lysates were extracted by incubation with 0.1 M NaOH at room temperature for 5 minutes. Lysates were mixed with SDS-sample buffer, boiled for 5 minutes, and subjected to Tris-HCl SDS-PAGE (4%–20% gradient, Bio-Rad), followed by transfer to a PVDF membrane (MilliporeSigma). Membranes were blocked in Odyssey blocking buffer (LI-COR) for 1 hour at room temperature. Primary antibody incubations were performed at 4°C overnight. After washing with PBS with 1% Tween (PBST), membranes were incubated with fluorescently labeled secondary antibodies at room temperature for 1 hour, followed by washing with PBST. Proteins were detected using an Odyssey Fc dual-mode imaging system (LI-COR). Antibodies used were rabbit polyclonal anti-GFP (MilliporeSigma, catalog G1544), mouse monoclonal anti-PGK1 (Thermo Fisher Scientific, catalog 459250), and fluorescently labeled anti-mouse and anti-rabbit secondary antibodies (LI-COR, catalog nos. 926-32210 and 926-68071, respectively).

### Yeast transformation and spotting assays.

All experiments were performed using *Saccharomyces* *cerevisiae* strain BY4741 (MATa, his3Δ1, leu2Δ0, met15Δ0, uraΔ30) (80). The PEG/lithium acetate method was used to transform yeast with plasmid DNA (81). For spotting assays, yeast cells were grown overnight at 30°C in liquid media containing raffinose (SRaf/-Ura) until they reached log or midlog phase. Cultures were then normalized for OD_600_; serially diluted; spotted onto synthetic solid media containing glucose, sucrose/galactose (1:1), or galactose lacking uracil; and were grown at 30°C for 2–3 days. Yeast experiments were performed in triplicate.

### Protein purification.

hnRNPA1 proteins were purified as described (10, 82, 83). Protein was centrifuged at 16,100*g* for 10 minutes at 4°C to remove any aggregated material before use in fibrillization and LLPS assays. After centrifugation, the protein concentration in the supernatant was determined by Bradford assay (Bio-Rad).

### In vitro fibrillization assays.

hnRNPA1 (5 μM) fibrillization (100 μL reaction) was initiated by addition of 2 μg of TEV protease in A1 assembly buffer (40 mM HEPES-NaOH at pH 7.4, 150 mM KCl, 5% glycerol, 1 mM DTT, and 20 mM glutathione). hnRNPA1 fibrillization reactions were incubated at 25°C for 24 hours with agitation at 1200 rpm in an Eppendorf Thermomixer, at which time fibrillization was complete with approximately 100% of the hnRNPA1 in the aggregated state. For sedimentation analysis, at indicated times, fibrillization reactions were centrifuged at 16,100*g* for 10 minutes at 4°C. Supernatant and pellet fractions were resolved by SDS-PAGE and stained with Coomassie Brilliant Blue, and the relative amount in each fraction was determined by densitometry in ImageJ (NIH). Fibrillization assays were performed in triplicate. For electron microscopy, fibrillization reactions (10 μL) were adsorbed onto glow-discharged 300-mesh Formvar/carbon-coated copper grids (Electron Microscopy Sciences) and stained with 2% (w/v) aqueous uranyl acetate. Excess liquid was removed, and grids were air-dried. Samples were viewed using a JEOL 1010 transmission electron microscope.

### In vitro LLPS assays.

hnRNPA1 liquid droplets were formed by incubating hnRNPA1 in the absence of TEV protease at the indicated concentration in LLPS buffer (40 mM HEPES-NaOH at pH 7.4, 150 mM NaCl, 5% glycerol, 1 mM DTT, and 20 mM glutathione, 10% dextran) at 25°C. Protein samples were spotted onto a coverslip immediately after all components had been added and imaged using a Leica DMI6000 by DIC microscopy.

### Immunofluorescence.

HeLa cells were seeded on 8-well glass slides (MilliporeSigma). Cells were transfected 24 hours after seeding using FuGENE 6 (Promega) with EGFP-tagged or enhanced yellow fluorescent protein–tagged (EYFP-tagged) hnRNPA1 constructs. Cells were stressed 24 hours after transfection with 500 μM sodium arsenite (MilliporeSigma) for 30 minutes. Cells were then fixed with 4% paraformaldehyde (Electron Microscopy Sciences), permeabilized with 0.5% Triton X-100, and blocked in 5% bovine serum albumin. Primary antibodies used were against G3BP1 (611127; BD Biosciences) and eIF3η (sc-16377; Santa Cruz Biotechnology). For visualization, the appropriate host-specific Alexa Fluor 555 or 647 (Molecular Probes, catalog no. A31570 or A21447, respectively) secondary antibody was used. Slides were mounted using ProLong Gold Antifade Reagent with DAPI (Life Technologies). Images were captured using a Leica TCS SP8 STED 3X confocal microscope (Leica Biosystems) with a 63× objective. Images were quantified by automated puncta analysis using CellProfiler software (Broad Institute). Cells were segmented using DAPI and G3BP1 channels, and granules were identified using G3BP1 and eIF3η channels. Integrated intensities of nucleus, cytoplasm, and granules were measured. Yeast cells that had been transformed with the indicated EGFP-tagged hnRNPA1 protein or vector were grown for 8 hours in galactose-containing media, pelleted, and imaged using a Leica DM IRBE microscope before being processed using ImageJ (NIH).

### Live-cell time-lapse imaging.

tdTomato-G3BP1–knockin U2OS cells were seeded in glass-bottomed, poly-d-lysine–coated, 35 mm dishes (MatTek Corporation). Cells were transfected 24 hours after seeding using FuGENE 6 (Promega) with EGFP-tagged or EYFP-tagged hnRNPA1 constructs. The dish was transferred 48 hours after transfection to an Opterra II swept-field confocal microscope (Bruker) system with a stage top incubator with 5% CO_2_ and 60× objective with an objective heater (Bioptechs), preheated to 37°C. The system was left to equilibrate for 5–10 minutes before imaging was initiated.

For time-lapse imaging, using PrairieView software with perfect focus engaged, multipoint images were taken every 30 seconds with both 488 and 560 nm lasers. Two minutes into imaging, the objective temperature was raised to 43°C to begin heat shock; 30 or 60 minutes later, the temperature was lowered back to 37°C to alleviate stress, and cells were imaged until granules disappeared, or after 2–3 hours passed. Cells with equivalent expression levels of hnRNPA1 were selected for further analysis.

### Live-cell analysis.

Live-cell imaging analysis was performed manually. Only hnRNPA1-positive viable cells that did not have td-Tomato G3BP1-positive granules prior to the 2-minute mark were evaluated. Cells were considered granule negative at the beginning of the video and were considered granule positive at the frame in which distinct cytoplasmic granules were visible. The frame at which cytoplasmic granules were no longer visible was defined as the time point at which cells were again SG negative. Images were analyzed using ImageJ (NIH). The percentage of cells that were considered granule positive was determined and graphed at each time point.

### Statistics.

Statistical analysis was performed in GraphPad Prism. Comparisons of the mean of each group with the mean of a control group were performed by 1-way ANOVA with Dunnett’s multiple comparison test. For comparing mean of control group with the means of other groups in each time point, 2-way ANOVA with either Sidak’s or Dunnett’s multiple-comparison test was performed. *P* values were considered significant at values less than 0.05 unless otherwise stated.

### Study approval.

All patients or their legal representatives signed informed consent forms before enrollment. The ethics review boards of the University of Antwerp and Antwerp University Hospital; the Mossakowski Medical Research Centre of the Polish Academy of Sciences; the University Hospitals St-Luc of the University of Louvain; Radboud University Medical Center; the Leiden University Medical Center; Stanford University; the University of North Carolina at Chapel Hill; and Antwerp University Hospital approved this study.

## Author contributions

AK, ND, PVDB, NCV, JBS, MTW, SP, PDJ, JB, and DK assessed the patients’ phenotypes. D. Beijer, IM, TD, KVS, KD, RJLFL, SMVDM, D. Bonner, and AM analyzed the genetic data. HJK, LG, JS, and JPT designed experiments, and D. Beijer, HJK, LG, KO, IM, TD, CMF, LED, and AFF performed experiments. D. Beijer, HJK, JS, JPT, and JB provided critical advice and analyzed the data. D. Beijer, HJK, CMF, JS, JPT, and JB wrote the manuscript. PDJ, JS, JPT, and JB conceived the project.

## Supplementary Material

Supplemental data

Supplemental Table 1

## Figures and Tables

**Table 1:**
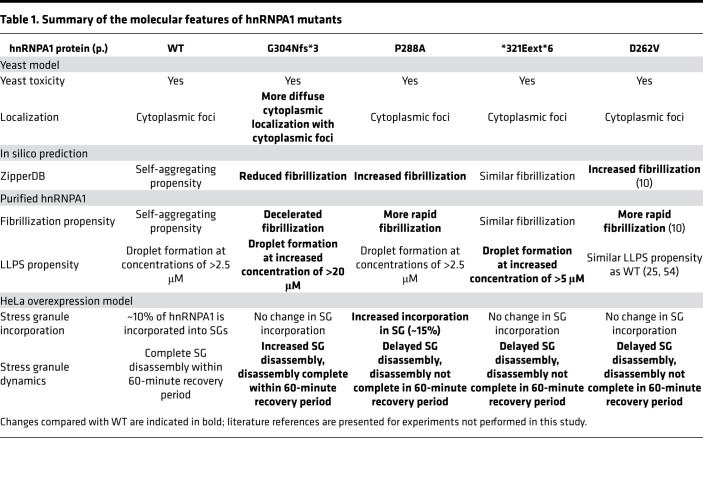
Summary of the molecular features of hnRNPA1 mutants

## Version 1 07/22/2021

Electronic publication
