# Supplementary material for: Characterization of HNRNPA1 mutations defines diversity in pathogenic mechanisms and clinical presentation
Source: JCI Insight. 2021 Jul 22;6(14):e148363. doi: 10.1172/jci.insight.148363 (PMC8410042; doi:10.1172/jci.insight.148363)
Supplement: Supplemental data [file jciinsight-6-148363-s052.pdf]

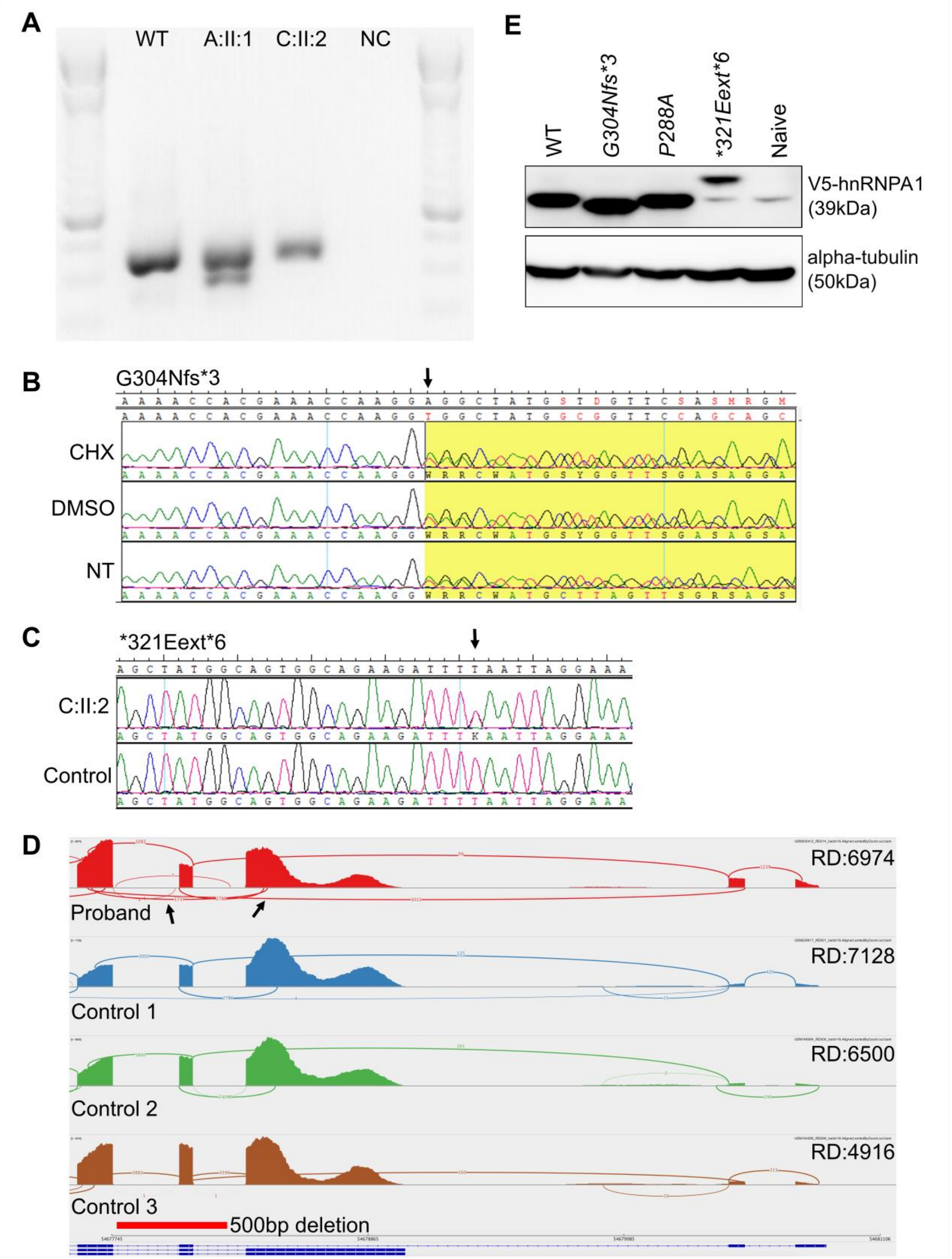

Fig. A1 Validation of HNRNPA1 mutations on mRNA level and splicing, as well as protein expression in HeLa overexpression model **A.** cDNA PCR results on agarose gel showing presence of shorter transcript in family A in addition to the wild-type band. Second transcript for family C is not visible on gel. **B.** cDNA sequencing at mutation site for the G304fs*3 mutation in family A, showing the mutation in heterozygous state in all three conditions (cycloheximide CHX, vehicle control DMSO and non-treated NT), thus not subjected to nonsense-mediated decay. **C.** Sanger sequencing of cDNA of patient C:II:2 and a healthy control, showing the presence of the *321Eext*6 mutation on mRNA level **D.** Sashimi plot demonstrating abnormal skipping of exon 9 NM_002136.4 (indicated by arrows) in D:II:1 proband (top row) when compared to control samples. Read depth (RD) listed per sample. The 500bp deletion shown as relative to the genomic location and NM_002136.4 transcript **E.** Western blot results of transfected HeLa cells with HNRNPA1-constructs showing bands of the expected size for all mutants. Wild-type HNRNPA1-V5 results in a band of approximately 39kDa, as does the P288A mutant. Whereas the G304Nfs*3 and the *321Eext*6 mutations result in a slightly smaller and slightly larger protein respectively.

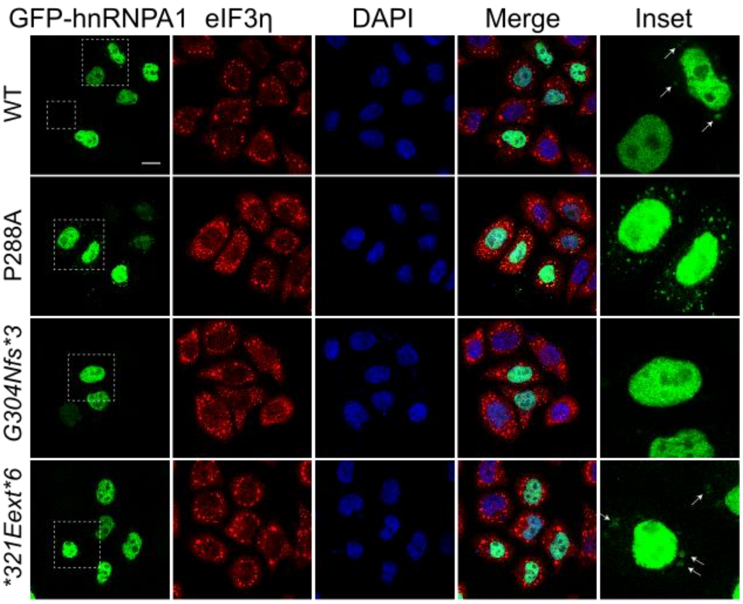

Fig. A2 Localization of the hnRNPA1 mutants subjected to heat shock

HeLa cells were transiently transfected with WT or mutant EGFP-tagged hnRNPA1 and subjected to heat shock treatment (temperature change from 37 ºC to 43 ºC for 30 min). Cells were fixed and stained with eIF3η (red) and DAPI (blue). Confocal images were taken for partition coefficient analysis, scale bar indicates 10 μm.

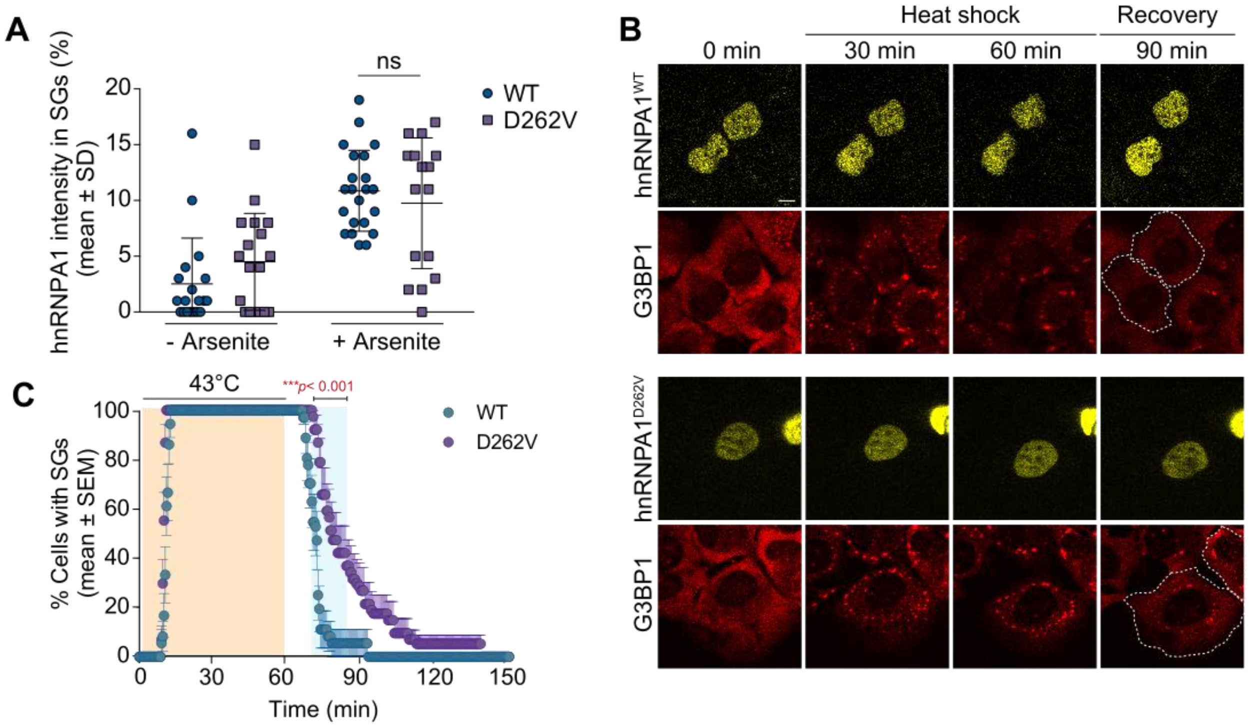

Fig. A3 Localization and stress granule dynamics of the hnRNPA1 D262V mutant

A. HeLa cells were transiently transfected with WT or mutant EGFP-tagged hnRNPA1 and subjected to arsenite stress (0.5 mM sodium arsenite, 30 min). Cells were fixed and stained with DAPI, eIF3η, and G3BP, and the intensity of hnRNPA1 signal in stress granules in each cell was measured. An interleaved scatter plot with individual data points is shown. Error bars represent mean ± SD (n = 18 and 22 cells for hnRNPA1 and hnRNPA1^{D262V}, respectively). ns, not significant by two-way ANOVA with Sidak's multiple comparisons test. Scale bar, 10 µm. **B,C.** U2OS cells expressing tdTomato-tagged endogenous G3BP1 were transiently transfected with WT or mutant EYFP-tagged hnRNPA1 and subjected to heat shock (43 °C, 60 min; orange shading) and allowed to recover at 37 °C for 2 h. White dotted lines delineate hnRNPA1-positive cells. Line graph in **c** represents the percentage of cells with visible tdTomato-G3BP1 puncta over time (n = 18 and 19 videos for hnRNPA1 and hnRNPA1^{D262V}, respectively). Blue shaded area indicates time points at which D262V mutant was statistically significantly different from WT. ***p < 0.001, by two-way ANOVA with Sidak's multiple comparisons test. Scale bar, 10 µm.

Table A1 Overview of genetic methods used for generation and analysis of NGS data

	Family A	Family B	Family C	Family D	Family E	Family F
**NGS type**	WES	WES	WGS	WGS	WES	WES
**Individuals with NGS**	A:II:1	B:II:1, B:II:2	C:I:1, C:I:2, C:II:2, C:III:1	D:II:1, D:I:1, D:I:2	E:I:2, E:II:1	F:I:1
**Exome capturing kit**	Nextera Rapid Capture Expanded Exome kit (62Mb) (Illumina)	SeqCap EZ Human Exome Library v3.0 (64Mb) (Roche)	-	-	SureSelect Clinical Research Exome V2 (Agilent)	Twist Human Core Exome kit + additional probes for human RefSeq transcripts (Twist Bioscience)
**Sequencing platform**	HiSeq 2500 (Illumina)	NextSeq 500 (Illumina)	HiSeq 2500 (Illumina)	HiSeq X (Illumina)	NovaSeq 6000 (Illumina)	NextSeq 500/550 (Illumina)
**Sequence alignment**	Burrows-Wheeler Aligner (BWA) (83)	Burrows-Wheeler Aligner (BWA) (83)	Burrows-Wheeler Aligner (BWA) (83)	Burrows-Wheeler Aligner (BWA) (83)	Burrows-Wheeler Aligner (BWA) (83)	Burrows-Wheeler Aligner (BWA) (83)
**Reference genome**	hg19, UCSC Genome Browser	hg19, UCSC Genome Browser	hg19, UCSC Genome Browser	hg19, UCSC Genome Browser	hg19, UCSC Genome Browser	hg19, UCSC Genome Browser
**Variant calling software**	Genome Analysis Toolkit (GATK) Unified Genotyper (85)	Genome Analysis Toolkit (GATK) Unified Genotyper and SAMtools (85, 86)	Genome Analysis Toolkit (GATK) Unified Genotyper (85)	Genome Analysis Toolkit (GATK) Unified Genotyper (85); Larger structural variant calling was performed using Manta (84)	Genome Analysis Toolkit (GATK) Unified Genotyper (85)	Genome Analysis Toolkit (GATK) Unified Genotyper (85)
**Analysis/filtering software**	Clinical Sequence Analyzer and Miner (Wuxi NextCODE)	GenomeComb (87)	Clinical Sequence Analyzer and Miner (Wuxi NextCODE)	In-house developed software, Codicem.	Illumina DRAGEN Bio-IT Platform v.2.03 and PerkinElmer's internal ODIN v.1.01 software	VariantDB (88)
**Filtering criteria**	Frequency ≤ 0.5% of the variants in public exome variant repositories (Exome Aggregation Consortium, 1000 Genomes Project, Exome Variant Server, in-house data); variants with impact on the encoded protein (missense, nonsense, frame shift, in-frame insertions/deletions and splice site variants); read depth ≥ 7; minimal heterozygous call percentage ≥ 20%	Frequency ≤ 0.5% of the variants in public exome variant repositories (Exome Aggregation Consortium, 1000 Genomes Project, Exome Variant Server, in-house data); variants with impact on the encoded protein (missense, nonsense, frame shift, in-frame insertions/deletions and splice site variants); read depth ≥ 7; minimal heterozygous call percentage ≥ 20%	Frequency ≤ 0.5% of the variants in public exome variant repositories (Exome Aggregation Consortium, 1000 Genomes Project, Exome Variant Server, in-house data); variants with impact on the encoded protein (missense, nonsense, frame shift, in-frame insertions/deletions and splice site variants); read depth ≥ 7; minimal heterozygous call percentage ≥ 20%	Exclusion of variants with low quality, present on an internally curated red herring list, within known polymorphic repeats that are not disease associated, <15% of reads supporting the variant call; Exclusion of variants with a MAF >5% according to ExAC, TGP, or gnomAD (unless they were identified as pathogenic or likely pathogenic in ClinVar, or identified as high confidence disease associated variants in HGMD; Inclusion of variants with impact on the encoded protein (missense, nonsense, frame shift, in-frame insertions/deletions and splice site variants)	No filtering, all sequence variants are assigned an interpretation category (Pathogenic, Likely Pathogenic, Variant of Uncertain Significance, Likely Benign and Benign) per ACMG Guidelines (89)	Frequency ≤ 2% of the variants in public exome/genome variant repositories (Genome Aggregation Database, gnomAD)); variants with impact on the encoded protein (missense, nonsense, frameshift, inframe insertions/deletions, splice site, start and stop loss variants); read depth ≥ 5; minimal allelic ratio ≥ 15%.<br>Analysis of variants was limited to genes listed in a predefined myopathy gene panel supplemented with genome-wide analysis based on HPO term associations (MOON software, Diploid/Invitae).
**Sanger sequencing confirmation**	Yes, in all available individuals	Yes, in all available individuals	Yes, in all available individuals	Yes, in all available individuals		Only for variants read depth < 30 or allelic ratio <40% or >60% for heterozygous variants.

84. Li H, and Durbin R. Fast and accurate long-read alignment with Burrows-Wheeler transform. *Bioinformatics.* 2010;26(5):589-95.

85. Chen X, Schulz-Trieglaff O, Shaw R, Barnes B, Schlesinger F, Kallberg M, et al. Manta: rapid detection of structural variants and indels for germline and cancer sequencing applications. *Bioinformatics.* 2016;32(8):1220-2.

86. McKenna A, Hanna M, Banks E, Sivachenko A, Cibulskis K, Kernytsky A, et al. The Genome Analysis Toolkit: a MapReduce framework for analyzing next-generation DNA sequencing data. *Genome Res.* 2010;20(9):1297-303.

87. Li H, Handsaker B, Wysoker A, Fennell T, Ruan J, Homer N, et al. The Sequence Alignment/Map format and SAMtools. *Bioinformatics.* 2009;25(16):2078-9.

88. Reumers J, De Rijk P, Zhao H, Liekens A, Smeets D, Cleary J, et al. Optimized filtering reduces the error rate in detecting genomic variants by short-read sequencing. *Nat Biotechnol.* 2011;30(1):61-8.

89. Vandeweyer G, Van Laer L, Loeys B, Van den Bulcke T, and Kooy RF. VariantDB: a flexible annotation and filtering portal for next generation sequencing data. *Genome Med.* 2014;6(10):74.

90. Richards S, Aziz N, Bale S, Bick D, Das S, Gastier-Foster J, et al. Standards and guidelines for the interpretation of sequence variants: a joint consensus recommendation of the American College of Medical Genetics and Genomics and the Association for Molecular Pathology. *Genet Med.* 2015;17(5):405-24.
