# Supplementary material for: Characterization of HNRNPA1 mutations defines diversity in pathogenic mechanisms and clinical presentation
Source: JCI Insight. 2021 Jul 22;6(14):e148363. doi: 10.1172/jci.insight.148363 (PMC8410042; doi:10.1172/jci.insight.148363)
Supplement: Supplemental Table 1 [file jciinsight-6-148363-s053.pdf]

Table 1 *HNRNPA1* mutations leading to a broad spectrum of phenotypes

Individual	A:II:1	B:II:1	B:II:2	C:II:2	C:III:1	D:II:1	E:II:2	F:I:1
**Gender**	Male	Female	Female	Female	Male	Female	Male	Male
**Parental consanguinity**	No	Yes	Yes	No	No	No	No	No
**Ethnicity**	Polish	Moroccan	Moroccan	Dutch	Dutch	Indian	Caucasian American	Belgian
**Current age (age at death)**	43 years	44 years	45 years	44 years	14 years	35 years	48 years	ł 64 years
**Mutation**								
**Genomic position (hg19)**	Chr12: 54678040 A>G	Chr12: 54677706 C>G	Chr12: 54677706 C>G	Chr12: 54678095 T>G	Chr12: 54678095 T>G	Chr12:54677766-54678265 deletion 500bp deletion	Chr12:54677629A >T	Chr12:54678095 T>C
**cDNA**	NM_002136: c.908-2A>G	NM_002136: c.862C>G	NM_002136: c.862C>G	NM_002136: c.961T>G	NM_002136: c.961T>G	NM_002136: c.907+15_*5-68del	NM_002136: c.785A>T	NM_002136: c.961T>C
**Protein**	p.G304Nfs*3	p.P288A	p.P288A	p.*321Eext*6	p.*321Eext*6	p.G304Nfs*3	p.D262V	p.*321Qext*6
**Clinical features**								
**Overall phenotype**	Distal pure motor neuropathy	Motor neuron disease	Motor Neuron disease	Distal myopathy	Distal myopathy	Distaly myopathy	Distal myopathy	Distal myopathy
**Age at onset**	15 years	22 years	43 years	9 years	8 years	22 years	36 years	12 years
**Symptoms at onset**	Wasting hand intrinsics	Paresis of left hand without sensory impairment	Swallowing difficulties and dysarthria	Difficulties with hand function, mild facial weakness, fatigue at age 12	Foot drop R	Hand weakness while writing	Weakness foot R frequent tripping and falls	Wasting of hand intrinsics, weakness lower limbs
**Age at last examination**	40 years	43 years	44 years	43 years	13 years	33 years	48 years	64 years
Walking (canes, wheelchair,….)	Ambulant	Ambulant	Ambulant	Wheelchair use: intermittent (28y) permanent (34y)	Ambulant	Ambulant	Cane, hand rails when going up the stairs	Wheelchair bound
Walking on toes	Yes	Yes	Yes	No	Yes	No	Yes	No
Walking on heels	No	Yes	yes	No	No	No	No	No
**Foot deformities**	Slight *pes cavus*	-	No	Slight hammer toes	*Pes planus* on right foot	No	High arched feet, hammer toes	*Pes equinovari*

**Muscle atrophy UL**	Hand intrinsics, distal forearm	R>L	Hand intrinsics R	Generalized	Thenar L	Hand intrinsics R>L	No	Pronounced
**Muscle atrophy LL**	Yes	No	No	Generalized	No	Yes	No	Pronounced
**Weakness proximal UL**	Not measured	Deltoid >biceps and triceps +++ bilaterally, neck flexors and abdominals	No	Shoulder abduction 2/5, Shoulder anteflexion mild	No	No	No	Biceps 0/5, triceps 2-/5, shoulder abduction 1-3/5
**Weakness distal UL**	Not measured	Hand intrinsics +++ bilaterally	Weakness (2/5) right abductor pollicis brevis and I interosseous	Hand intrinsics 2/5, finger flexion/extension 0/5	Opponens pollicis MRC 5-/5 L	finger and wrist extension 1/5 R , 4-4+/5 L	No	0/5
**Weakness proximal LL**	Not measured	No	No	Knee extension 1/5, hip adduction/ abduction 1/5	No	No	Knee flexion/ extension 4+/5	0/5
**Weakness distal LL**	Not measured	No	No	0/5	Foot plantar flexion 5/5, Foot/toe dorsiflexion 3/5 L, 0/5 R	ankle dorsiflexion 0/5 R, 4-/5 L	Foot eversion 4+/5, inversion 5/5, dorsiflexion 3+/5, plantar flexion 4-/5	tibialis anterior 2/5, otherwise 0/5
**Reflexes UL**	Very brisk	Very brisk	Brisk	Absent	Normal	Trace	Normal	Absent
**Reflexes LL**	Brisk	Absent	Normal	Absent	Normal	Trace	Normal	Absent
**Predominance in the hands**	Yes	Yes but also severe in deltoids	Yes	Yes	No	Yes	No	Yes (initial presentation)
**Sensory involvement**	-	-	-	-	-	No	No	No
**Pyramidal tract signs (Babinski sign, clonus, …)**	No	Bilateral Hoffmann-Trömner, no Babinski sign	Right Hoffmann-Trömner, no Babinski sign	No	No	No	No	No
**Respiratory difficulties**	No	Vital capacity 59%	No	Vital capacity 70%, aggravated during cold or fever	No	Forced vital capacity 76%	No	Non-invasive ventilation (BiPAP), cough assist
**Facial weakness**	No	Yes	No	Yes, very pronounced	No	Yes	No	Yes, very pronounced

**Other clinical features**	Mild thoracic scoliosis, depression, mild deformation of the L1 and L2 vertebrae	-	Tongue atrophy and dysarthria	Dysarthria, most likely due to the very severe facial weakness	-	Asymmetry of weakness, scapular winging	-	Percutaneous endoscopic gastrostomy, muscle contractures UL and LL, dysphagia, titubation
**Additional testing**								
**NCS**	Pure motor axonal neuropathy	Pure motor axonal neuropathy	-	Pure motor axonal neuropathy	Normal	Decreased amplitude median and peroneal motor	low amplitude peroneal motor consistent with chronic myopathy	Normal, very small CMAPs
**Needle EMG**	Chronic neurogenic	Not performed	Not performed	Myopathic, proximal UL	Myopathic, distal and proximal	Irritative distal predominant myopathy.	Chronic myopathic changes with muscle membrane irritability in LL	Chronic myopathic
**Nerve biopsy**	Very mild axonal neuropathy with secondary demyelination (25y)	Not performed	Not performed	Not performed	Not performed	Not performed	Not performed	Normal
**Muscle biopsy**	Not performed	Not performed	Not performed	Angular fibers, no type grouping (17y)	Slight clustering type 1 fibers (8y)	Myofibrillar disorganization, rimmed vacuoles no inflammation (33y)	Chronic myopathy with rimmed vacuoles (45 years)	Type 1 atrophy, limited rimmed vacuoles, centralized nuclei type 2 fibers (13y)

L, left: LL, lower limb(s): MRC, medical research council: NCS, nerve conduction studies: R, right: UL, upper limb(s): (-), absent/unknown
